# The Role of Nanomaterials in Stroke Treatment: Targeting Oxidative Stress

**DOI:** 10.1155/2021/8857486

**Published:** 2021-03-17

**Authors:** Guini Song, Min Zhao, Hanmin Chen, Cameron Lenahan, Xiangyue Zhou, Yibo Ou, Yue He

**Affiliations:** ^1^Department of Neurology, Tongji Hospital, Tongji Medical College, Huazhong University of Science and Technology, Wuhan, Hubei, China; ^2^Department of Neurosurgery, Tongji Hospital, Tongji Medical College, Huazhong University of Science and Technology, Wuhan, Hubei, China; ^3^Department of Biomedical Sciences, Burrell College of Osteopathic Medicine, Las Cruces, NM, USA

## Abstract

Stroke has a high rate of morbidity and disability, which seriously endangers human health. In stroke, oxidative stress leads to further damage to the brain tissue. Therefore, treatment for oxidative stress is urgently needed. However, antioxidative drugs have demonstrated obvious protective effects in preclinical studies, but the clinical studies have not seen breakthroughs. Nanomaterials, with their characteristically small size, can be used to deliver drugs and have demonstrated excellent performance in treating various diseases. Additionally, some nanomaterials have shown potential in scavenging reactive oxygen species (ROS) in stroke according to the nature of nanomaterials. The drugs' delivery ability of nanomaterials has great significance for the clinical translation and application of antioxidants. It increases drug blood concentration and half-life and targets the ischemic brain to protect cells from oxidative stress-induced death. This review summarizes the characteristics and progress of nanomaterials in the application of antioxidant therapy in stroke, including ischemic stroke, hemorrhagic stroke, and neural regeneration. We also discuss the prospect of nanomaterials for the treatment of oxidative stress in stroke and the challenges in their application, such as the toxicity and the off-target effects of nanomaterials.

## 1. Introduction

Stroke is a disease associated with substantial morbidity and disability. It is a leading cause of death and is associated with a 24.9% lifetime risk of stroke (18.3% for ischemic stroke and 8.2% for hemorrhagic stroke) for global populations over 25 years old [1, 2]. Ischemic stroke is primarily caused by thrombosis or embolism, which leads to a lack of blood and oxygen in the brain. However, the arteriosclerosis and aneurysms are common etiologies of hemorrhagic stroke [3–5]. After stroke, overproduction of reactive oxygen species (ROS) is a critical mechanism responsible for brain injury [6–8]. Excessive ROS can react with lipid membranes, proteins, and nucleic acids, which causes cellular apoptosis and cell death in the brain [7]. However, antioxidant therapy in stroke has made little advancement in previous years. The difference between preclinical studies and clinical studies about the antioxidant therapy for stroke may be related to several factors, including the antioxidants' half-life and differences of the blood-brain barrier (BBB) between human and rodents. This proposes a great challenge for clinical translation of antioxidant therapy in stroke [9, 10].

Nanotechnology is an emerging field that can greatly complement medical therapy. It comprises the design, synthesis, and application of nanomaterials for the treatment of diseases and takes advantages of materials at the atomic and molecular scale [11, 12]. Nanomaterials can exist in many shapes, such as spheres, dots, platelets, tubes, dendrites, and rods. Meanwhile, they can be neutral, positively or negatively charged [12]. Nanomaterials can provide new diagnostic and treatment methods for medicine [13] and may be considered an advanced approach in the antioxidant therapy of stroke due to their unique features, such as small size and stability, as well as their long serum half-life [14, 15]. These nanomaterials assist antioxidants in crossing the BBB and providing a protective shell for antioxidants. Additionally, some nanomaterials, such as platinum nanoparticles (PtNPs) and cerium oxide nanoparticles (nanoceria), have an antioxidant effect [16, 17], which can be beneficial for the treatment and recovery of stroke. The application of nanomaterial has shown great promise in stroke antioxidant treatment and recovery.

## 2. Nanomaterials

Nanomaterials are usually 1-500 nm in diameter and are easily taken up by cells. The smaller the nanomaterial, the larger the surface area to volume ratio, means an increased efficiency of interaction with tissue cells [18]. Additionally, nanomaterials can protect antioxidants from decomposition, which would extend the serum half-life [19, 20]. The biological half-life of nanomaterials is related to their design (e.g., size and shape) and surface modification [21]. Surface modification, such as PEGylation, is related to the biocompatibility of nanomaterials, as it can deliver the antioxidants to the brain tissue and reduce liver metabolism, mononuclear phagocytic system (MPS) uptake, and kidney clearance of nanomaterials, but may improve the bioavailability of antioxidants [22]. PEGylation, the combination of polyethylene glycol (PEG) and nanomaterials, can increase the hydrophilicity and stability of nanomaterials [23]. The surface properties of nanomaterials, such as hydrophobicity or hydrophilicity, allow them to carry the corresponding compounds [24]. Because of their small size, nanomaterials have obvious advantages in passing through natural barriers, such as the BBB. They can reach the damaged site quickly and may accumulate in brain tissue [25].

The BBB is composed of vascular endothelial cells, astrocytes, pericytes, and the basement membrane and is responsible for regulating the exchange of substances between blood and the cerebrospinal fluid (CSF). The thickness of the endothelial cells at BBB in rodents is only 150–240 nm. Meanwhile, the thickness of human endothelial cells is between 370 and 420 nm [26], which provides a substantial barrier to the diffusion and transport of therapeutic molecules. Nearly 98% of small molecules and all large molecule drugs cannot cross the BBB [27]. Conversely, nanomaterials can pass through biological membranes in two ways: active transport and passive transport, which is dependent on the size, shape, surface characteristics (e.g., hydrophilicity or lipophilicity), and the surface modification of nanomaterials. Passive transport is commonly used in cancer. Nanomaterials can cross endothelial cells through the enhanced permeability and retention effect when there is increased microvasculature permeability in cancer [28]. Therefore, polymeric nanoparticles with larger diameters pass through the BBB primarily via transcytosis. To increase the transcytosis of nanomaterials, ligands on the surface of nanomaterials can be modified for specific receptors on the BBB, and nanomaterials can be mediated by ligand-receptor binding to pass through the BBB. These receptors include the transferrin receptor, insulin receptor, low-density lipoprotein receptor (LDLR), angiopep-2 receptor, and the receptor for advanced glycation end-products (RAGE) [29–33]. The modified ligands on nanomaterials can allow them to cross the BBB efficiently.

The following paragraph will talk about the variety of nanomaterials that have been used for antioxidant therapy in stroke, including metallic nanoparticles and metal oxide nanoparticles, carbon-based nanoparticles, liposome nanoparticles, and polymeric nanoparticles.

### 2.1. Metallic Nanoparticles and Metal Oxide Nanoparticles

Metallic nanoparticles are nontoxic with good biocompatibility, and they can be modified to carry a variety of substances due to the negative charge on their surface. Because of the free electrons on the surface, some metallic and metal oxide nanomaterials, such as PtNPs and nanoceria, show strong ROS-scavenging activity with stable chemical properties [16, 34, 35]. The studies showed that PtNPs can mimic the activity of antioxidant enzymes, scavenge free radical, and transform superoxide anion (•O_2_^−^) into H_2_O and O_2_ [16, 36]. Nanoceria exist in both Ce^3+^ and Ce^4+^ oxidation states. Due to the oxygen vacancies on their surface, nanoceria can redox cycle between Ce^3+^ and Ce^4+^ states. Moreover, Ce^3+^ reacts with hydroxyl radicals (•OH) to generate Ce^4+^ and then generates Ce^3+^ and O_2_ under the action of H^+^, leaving oxygen vacancies in the nanomaterials. This allows nanoceria to exert their catalytic activity, imitating the properties of superoxide dismutase (SOD) and catalase (CAT) and converting •OH into O_2_ [17, 35]. The PEGylated nanoceria have colloidal stability and reduced agglomerations. Moreover, the catalytic properties of nanoceria are enhanced by the higher ratio of Ce^3+^, facilitating the creation of ultrasmall nanoceria [37]. Additionally, 3 nm nanoceria were synthesized through the aqueous phase and increased the ratio of Ce^3+^ in nanoceria to approximately 57% [38].

### 2.2. Carbon-Based Nanoparticles

Carbon-based nanoparticles commonly include fullerenes and carbon nanotubes (CNTs). Fullerenes, namely C_60_ nanoparticles, are spherical in shape, have abundant conjugated double bonds, and have the ability to absorb electrons. Therefore, they can perform the same function as SOD and scavenging free radicals [39]. Modification of fullerenes, such as polyhydroxylated fullerenes and carboxyfullerenes, can improve the stability of nanoparticles and allow them to localize in the mitochondria, leading to the protection of mitochondria and reduction of free radical generation [40, 41]. The antioxidant activity of fullerenes is related to its size, structure, and surface chemical properties. Different surface functional groups can exert different oxygen metabolism regulation, thereby increasing or reducing the production of ROS and exerting either a prooxidation or antioxidant effect [42]. A CNT is a chemically stable, cylindrical molecule composed of graphite. It has antioxidant activity and high conductivity, but it is not biodegradable in the body and easily forms agglomerates (large aggregates). Therefore, it requires surface modification. Amino-functionalized CNTs are degradable in mouse brain after postinjection; meanwhile, functionalization of CNTs using PEG, chitosan, bovine serum albumin (BSA), or surfactants increases their biostability and dispersibility and reduces aggregate formation and cytotoxicity [43, 44].

### 2.3. Liposome Nanoparticles

Liposome nanoparticles are composed of amphiphilic molecules similar to biological membranes. Therefore, liposomes have good biocompatibility and biodegradability. They can carry hydrophobic or hydrophilic drugs, improve drug efficacy, and reduce adverse reactions [45]. Liposomes have been used in clinical applications, such as delivering chemotherapeutic drugs in cancer treatments [46]. However, the disadvantages of liposomes include reduced drug packaging efficiency and quick drug release. Processes of liposomal modification, such as PEGylation, can extend the half-life of liposome nanoparticles, prevent leakage or fusion of nanodrug particles, and improve the stability and bioavailability of sensitive compounds [23, 47]. Additionally, liposomes can be used for targeted delivery of antioxidants. They can also be modified with ligands for receptor targeting, such as transferrin, and this leads to enhanced translocation across the BBB [48]. Additionally, echogenic liposomes (ELIP) can be guided by ultrasound to the target site [49].

### 2.4. Polymeric Nanoparticles

Polymeric nanoparticles are the most commonly used nanomaterials in drug delivery and are praised for their excellent biocompatibility and biodegradability. Polymeric nanoparticles are made of natural polymers (e.g., chitosan) or synthetic polymers (e.g., poly(lactic-co-glycolic acid) (PLGA), polylactide (PLA), poly(amidoamine) (PAMAM), or poly(methyl methacrylate) (PMMA)), and these materials have great surface modification potential and good pharmacokinetic characteristics [50, 51]. Micelles and dendrimers are commonly used in polymeric nanoparticles. The micelles have a hydrophilic outer shell and a hydrophobic inner core, which requires them to be manufactured by amphiphilic polymers [52]. Therefore, micelles can carry hydrophilic or hydrophobic drugs without changing the structure of the drugs [53]. PLGA is the most common type of polymeric nanoparticles, which is often spherical in shape. In addition, it is easy for processing and modification and can regulate stable drug release [54, 55].

## 3. Targeting Stroke Oxidative Stress Using Nanomaterials

### 3.1. Targeting Oxidative Stress in Ischemic Stroke

All the nanomaterials for the treatment of ischemic stroke were listed in Table 1. According to the route of antioxidant treatment, they can be divided into ROS scavenger nanomaterials, nanomaterials as carriers to transport free radical scavengers, to transport antioxidant enzymes, to transport antioxidant drugs, and to transport antioxidant genes.

#### 3.1.1. ROS Scavenger Nanomaterials

**Figure 1:**
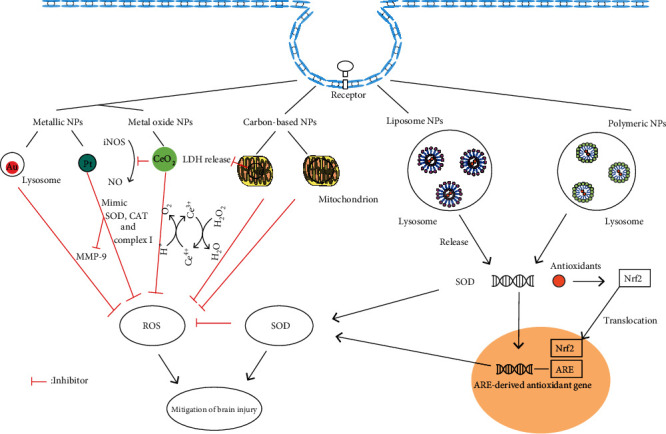
Antioxidant mechanism of nanoparticles in ischemia stroke. Nanoparticles enter cell by receptor-mediated endocytosis; based on their composition, nanoparticles can be divided to four main groups: metallic and metal oxide nanoparticles, carbon-based nanoparticles, liposome nanoparticles, and polymeric nanoparticles. Metallic and metal oxide nanoparticles and carbon-based nanoparticles exert free radical scavenging properties. AuNPs are found in both cytoplasm and lysosome. PtNPs mimic the activity of SOD, CAT, and mitochondrial complex I and decrease the ROS and MMP-9 activation. Ce^3+^ in nanoceria reacts with •OH to generate Ce^4+^ and then generates Ce^3+^ and O_2_ under the action of H^+^. Nanoceria downregulate iNOS to reduce the production of NO and eliminate ONOO^−^. Modification of C_60_ and HCCs allows them to be localized in mitochondria. Hexasulfobutylated C_60_ reduces LDH release. Meanwhile, PEG-HCCs exert functions as SOD. Liposome and polymeric nanoparticles can load antioxidants, antioxidant enzymes, and genes to reduce the free radical in stroke. Most of antioxidants activate Nrf2, promote Nrf2 translocation to nucleus, and bind with antioxidant response element (ARE) to promote the expression of ARE-derived antioxidant gene. These protect brain injury from stroke. NPs: nanoparticles; Au: gold nanoparticles; Pt: platinum nanoparticles; CeO_2_: nanoceria; HCC: hydrophilic carbon clusters; SOD: superoxide dismutase; CAT: catalase; MMP-9: matrix metalloproteinase-9; iNOS: inducible nitric oxide synthase; NO: nitric oxide; ONOO^−^: peroxynitrite; LDH: lactate dehydrogenase; Nrf2: nuclear factor-erythroid 2-related factor 2; ARE: antioxidant response element; ROS: reactive oxygen species; PEG: polyethylene glycol; •OH: hydroxyl radicals.

The application of nanomaterials regarding ROS scavengers in stroke has been extensively studied. Metallic nanoparticles performed an excellent antioxidant effect in stroke therapy (Figure 1). Treatment with PtNPs in transient middle cerebral artery occlusion (tMCAO) mice significantly reduced the infarct volume, matrix metalloproteinase-9 (MMP-9) activation, and •O_2_^−^generation [56, 57]. This may relate to PtNPs, which can serve the same function as the mitochondrial complex I [36]. Unlike PtNPs, gold nanoparticles (AuNPs) can exhibit either oxidative or antioxidant activity in stroke treatment, depending on the size of nanoparticles. Studies found that 20 nm AuNPs can reduce cerebral infarction in rats, while 5 nm AuNPs lead to enlarged infarction [58]. Further cell experiments revealed the same results and may be explained by the accumulation of 5 nm AuNPs into the nucleus, causing DNA damage [34].

Nanoceria reduce approximately 50% of ischemic cell death in the mouse hippocampal slice model of cerebral ischemia, in which the level of 3-nitrotyrosine decreased by approximately 70% [59]. Nanoceria downregulate inducible nitric oxide synthase (iNOS) to reduce the production of nitric oxide (NO) in mouse macrophages and eliminate peroxynitrite (ONOO^−^) generated by the reaction of •O_2_^−^ and NO [60, 61]. Meanwhile, nanoceria can polarize microglia into the M2 type and reduce oxidant-mediated cell apoptosis [62, 63]. Additionally, studies on stroke in rats have found that 0.5 and 0.7 mg kg^−1^ of nanoceria can eliminate ROS by 50%. However, 1.0 and 1.5 mg kg^−1^ of nanoceria failed to protect against stroke [37]. It may be related to excessive ROS elimination, which affects the signal transduction in cell [64]. Other modifications of nanoceria, such as bioactive zeolitic imidazolate framework-8 (ZIF-8), have also exerted protective effects in mouse cells [65].

Carbon-based nanoparticles also exert neuroprotective effect against oxidative stress (OS) and reduce the volume of cerebral infarction by 50% [66, 67]. Fullerene nanoparticles activate the c-Jun NH2 terminal protein kinase (JNK) in the brain microvascular endothelial cells and inhibit the cleavage of polyADP-ribose polymerase (PARP) to inhibit cell apoptosis [41]. Hexasulfobutylated C_60_ reduces lactate dehydrogenase (LDH) release in MCAO rats and increases NO content [66]. The injection of single-walled CNTs functionalized with PEG (SWCNT-PEG) in the hippocampus of normal rats showed an increase in the expression of antioxidant enzymes after an extended period of time [44]. PEGylated hydrophilic carbon clusters (PEG-HCCs) (HCC was generated by oxidation of SWCNT) exert functions as SOD [67–69] and can also scavenge •OH in the brain endothelial cell and primary cortical neuron cell [67]. PEG-HCC exhibits an estimated reduction potential similar to that of ubiquinone. Moreover, PEG-HCC has an improved protective effect against the H_2_O_2_ toxicity when compared with methylene blue, and it colocalizes in the mitochondria. Lastly, when using sodium cyanide to inhibit the mitochondrial complex IV in the cell, PEG-HCC demonstrated a protective effect on cells [69].

#### 3.1.2. Nanomaterials as Carriers to Transport Free Radical Scavengers

Free radical scavengers, such as 2,2,6,6-tetramethylpiperidine-1-oxyl (TEMPO), edaravone, vitamins E and C, and N-acetylcysteine (NAC), have shown promise in ischemic stroke [45, 70–72]. However, they have failed clinical trials in USA. This may result from short half-life [19, 20]. PEG-b-poly 4-amino-TEMPO aminomethylstyrene nanoparticles (nitroxide radical-containing nanoparticles [RNPs]) and micelle-encapsulated 4-amino-TEMPO nanoparticles in mice showed protective effects, and the half-life of RNPs is 60 times longer (15 minutes) than that of TEMPO [19, 70, 73]. The t-PA and 4-amino-TEMPO-containing self-assembled polyion composite nanoparticles (t-PA @ iRNP, which means iRNP containing t-PA) improved the half-life and bioavailability of t-PA compared with t-PA alone in MCAO mice. Furthermore, t-PA @ iRNP also reduces the hemorrhage induced by t-PA [74]. Monodisperse nanoceria are loaded with edaravone and modified with PEG and angiopep-2 on their surface to form EA/P-CeO_2_ and show synergistic scavenging activity of free radicals in both *in vivo* and *in vitro* models [31]. Additionally, agonistic micelles carrying edaravone, liposomes or PLGA carrying vitamins C and E, and PAMAM dendrimer or PLGA carrying N-acetylcysteine (NAC), all demonstrated good stability and antioxidant activity [20, 45, 54, 72, 75].

#### 3.1.3. Nanomaterials as Carriers to Transport Antioxidant Enzymes

Nanomaterials may be used as carriers for antioxidant enzymes. Antioxidant enzymes are capable of scavenging more ROS in stroke. Natural antioxidant enzymes remain in the blood for approximately 6 minutes and then quickly degrade in the serum, as it is difficult to cross the BBB [55]. PLGA-coated SOD can reduce the infarct area by 65% during rat ischemia/reperfusion and has a better survival rate at 28 days [55, 76]. This is obviously related to the increased bioavailability of SOD as a result of the long half-life. SOD1 and human immunodeficiency virus (HIV) transactivator protein (TAT) form into a fusion protein, which can be loaded onto silica nanoparticles to penetrate the BBB by TAT [29]. SOD is loaded onto methoxy-PEG-poly(L-lysine hydrochloride), polyethyleneimine-polyethylene glycol (PEI-PEG) polymer, and nanosized polyion complexes or silica to facilitate the transportation and reduction of hydrolysis of antioxidant enzymes and increase the activity of scavenging ROS [77–80].

#### 3.1.4. Nanomaterials as Carriers to Transport Antioxidant Drugs

Nanomaterials transport and reduce the metabolism of antioxidants in the serum to target the ischemic area. Polyphenols and flavonoids, such as resveratrol, thymoquinone, and quercetin, have antioxidation capabilities through the activation of nuclear factor-erythroid 2-related factor 2 (Nrf2), peroxisome proliferator-activated receptor *γ* (PPAR*γ*), and forkhead box O (FoxO) [81, 82]. Thus, nanoparticle-loaded polyphenols have been widely studied. Resveratrol-loaded nanoparticles (RES-NPs) basing on poly(N-vinylpyrrolidone)-b-poly(*ε*-caprolactone) polymer (PVP-b-PCL) has biodegradability and high drug-loading capacity; mesoporous silica nanoparticles (MSNP) loaded resveratrol, modified with biodegradable ROS-sensitive PLA, and used a new peptide (Ac- [cMPRLRGC]c-NH2) as a ligand for LDLR to cross the BBB via transcytosis. Additionally, resveratrol polymer nanoparticles all exert protective effects against brain injury [30, 83, 84]. Thymoquinone nanoemulsion and PLGA-chitosan nanoparticles reduced lipid peroxidation when administered intranasally [85, 86]. Meanwhile, PLGA polymer-encapsulated quercetin can reduce neuronal damage in rats, but quercetin does not have effect on brain I/R damage, mainly due to hydrophobicity [87].

Other antioxidants carried by nanomaterials have also been studied. Panax notoginseng (PNS) is a traditional Chinese medicine with antioxidant properties. Zhang et al. [88] studied novel liposomal systems encapsulating methyl ether PEG-PLGA-based nanoparticles, namely, core-shell hybrid liposomal vesicles (HLVs). Compared with liposomes, the encapsulation efficiency and the stability of the HLVs are better and more suitable for oral administration. Lycopene liposomes reduce the levels of nitric oxide synthase (NOS) and NADPH oxidase 2 (NOX2) [89]. Acetyl-11-keto-*β*-boswellic acid o-carboxymethyl chitosan nanoparticles (AKBA-NP), gallic acid o-carboxymethyl chitosan nanoparticles (GA-NP), retinoic acid nanoparticles (RA-NP), erythropoietin (EPO) liposomes, and EPO-coated PLGA nanoparticles all show better neuroprotective effects compared with AKBA, GA, RA, and EPO alone, respectively [90–95].

Neutrophils migrate into injured tissue after ischemic stroke. Promotion of neutrophil clearance in the ischemic brain can attenuate the volume of cerebral infarction after tMCAO [96]. Tang et al. invented platelet-mimetic nanoparticles (PTNPs), which use piceatannol, a selective spleen tyrosine kinase (Syk) inhibitor, coloaded PLGA core, with a platelet membrane surrounding the core. Under the guidance of p-selectin, nanoparticles can specifically bind to neutrophils to internalize and release piceatannol, which effectively inhibits Syk phosphorylation and significantly alleviates neutrophil adhesion and migration, preventing neutrophilic infiltration into the ischemic tissue. It was found that PTNPs reduced the infarct area in MCAO mice, which provided a novel idea for the application of nanomaterials in OS [97].

#### 3.1.5. Nanomaterials as Carriers to Transport Antioxidant Genes

Nanomaterials can be used as gene delivery carriers to interfere with the OS in stroke. They have minimal cytotoxicity and higher transfection efficiency (Table 1). HSAP-NP/pHO1 micelles are created based on deoxycholate-conjugated polyethylenimine-2k (DP2k) and loaded with hypoxia-specific anti-RAGE peptide (HSAP) and the heme oxygenase-1 plasmid (pHO1); they are internalized by RAGE *in vivo* and deliver pHO1 to protect hypoxic cells and MCAO mice [32]. R3V6 peptide micelles, which have a strong hydrophobic core, are assembled by 3-arginine, 6-valine, and dexamethasone, had a high transfection efficiency; meanwhile, deoxycholic acid-conjugated PEI (DA-PEI) for delivery of heme oxygenase-1 gene achieved higher HO-1 expression [98, 99]. Reducible poly(oligo-D-arginines) (rPOA) were synthesized and demonstrated lower toxicity, but higher transfection efficiency than PEI [100]. Polyamidoamine generation 2 dendrimer (PG2) conjugated histidine and arginine, synthesized PG2HR, reduced cytotoxicity, demonstrated protective effects, and may also be an efficient gene carrier [101]. Therefore, nanomaterials can be a safe and biodegradable gene carrier for antioxidant therapy.

### 3.2. Targeting Oxidative Stress in Hemorrhage Stroke

Cerebral parenchymal and subarachnoid hemorrhages are the most common types of hemorrhagic strokes. A substantial amount of blood enters the brain, causing OS and nerve injury in intracerebral hemorrhage (ICH). Similar to ischemic stroke, antioxidants, such as deferoxamine (DEF), have not demonstrated significant effects in clinical trials [102]. Dharmalingam et al. [103] found that DEF and PEG-HCCs bound covalently to form the DEF-HCC-PEG and can reduce DNA damage response signaling, mitochondrial DNA damage, and ROS formation caused by heme in both *in vivo* and *in vitro* experiments, and the dosage of DEF-HCC-PEG is reduced by 200-300 times when compared with deferoxamine. Meanwhile, quercetin loaded nanoemulsions (which mainly use phospholipids as surfactants) with an entrapment efficiency of 98.4% and improved locomotor function compared with quercetin alone in an ICH rat model [104].

Subarachnoid hemorrhage (SAH), a dangerous type of stroke with a very high mortality rate, has an etiology of cerebral artery rupture [105]. Excessive ROS production in the early stage of SAH causes microcirculation dysfunction, leading to early brain injury (EBI) [106]. Antioxidant therapy is essential for SAH. Nanoceria were modified with aminocaproic acid and PEG, which reduced the apoptosis of macrophages and accumulation in the ipsilateral cerebral hemisphere where the aneurysm was ruptured [38]. Meanwhile, curcumin, encapsulated in PLGA nanoparticles and NO-loaded ELIP (NO-ELIP), extenuates EBI [49, 107]. Aneurysmal repair essentially functions as a prophylactic for future hemorrhages, and platinum coils are used in clinical practice. Pt-coated nanofibers (created via electrospinning and electroplating) show very low permeability and can be used as a substitute for platinum coils [108]. Although these Pt-coated nanofibers have not been evaluated for OS in blood vessels, they still have viable clinical potential. The antioxidant effect of nanomaterials in cerebral hemorrhage and subarachnoid hemorrhage has not been thoroughly studied, but it is undeniable that there are great therapeutic prospects.

Cerebral cavernous malformation (CCM) is a multifactorial disease that affects approximately 0.4–0.8% of the general population [109]. CCM is caused by CCM gene (CCM1, CCM2, and CCM3) mutations [110], which cause abnormally dilated capillaries and a risk of seizure and intracranial hemorrhage [109]. Meanwhile, it affects cellular redox homeostasis and autophagy, leading to mitochondrial dysfunction and increased ROS [111, 112]. PtNPs have been studied in mouse embryonic fibroblast (MEF) cells, which are derived from a CCM1 knockout mouse model that recapitulates the human CCM. They found that ROS levels are close to normal cells, which means that PtNPs recover cellular ROS homeostasis in MEF cells [16]. De Luca et al. [113] studied multifunctional platinum@BSA-rapamycin nanoparticles (Pt5@Rapa NPs), which consist of 5 nm PtNPs, rapamycin, and bovine serum albumin (BSA). These deliver rapamycin to lysosomes in MEF cells, modulate ROS homeostasis and angiogenesis, and achieve maximum synergy in treatments. Studies have shown the effect of nanomaterials on CCM *in vitro*, but these effects on CCM must be studied *in vivo*. Multifunctional nanocarriers in combinatorial treatments of CCM warrant further investigation.

### 3.3. Targeting Oxidative Stress in Neural Regeneration

**Figure 2:**
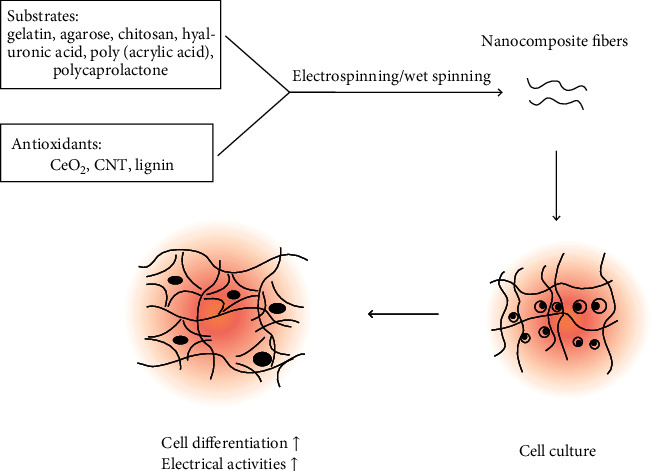
Schematic representation of nanocomposite formation mechanisms and their antioxidant effects in neuronal regeneration. Nanocomposite fibers are prepared by substrates and antioxidants, using electrospinning or wet spinning, and promote cell differentiation and electrical activities in neuron culture. CeO_2_: nanoceria; CNT: carbon nanotube.

Neural regeneration after stroke is related to the prognosis. Moreover, OS and the inflammatory environment after nerve injury cause secondary damage to the nerve, leading to the death of the neural network. Studies have found that antioxidants can promote regeneration after nerve injury [114]. Nanoscaffolds used in nerve repair can provide a microenvironment for cell attachment and can guide cell growth and imitate the extracellular matrix of neurons for tissue repair [115, 116] (Figure 2). The electrospun nanofiber scaffold, modified with 10 nm AuNPs, promoted immature neurons to grow axons more than branched trees [117]. Nanoceria fibers (synthesized by nanoceria and gelatin) promote *β*3-tubulin mRNA expression (related to neuronal differentiation) and axonal growth, as well as demonstrate improved neuron electrical activity [118–120]. However, Gliga et al. reported a contradictory result [121]. This may be related to the differences in sizes, doses, modification, and ratios of Ce^3+^ to Ce^4+^, but nanoceria in low ratios of Ce^3+^ to Ce^4+^ promote cell proliferation [122, 123]. Carbon-based nanoparticles, such as agarose CNT fibers, promote cellular adhesion and neuronal differentiation and can be used for neural tissue engineering [124–126]. Anti-transferrin receptor monoclonal antibody-PEGylated Se nanoparticles (PEG-Se NPs) and lignin-polycaprolactone copolymer nanofiber scaffolds also promote cell proliferation [127, 128]. Meanwhile, gelatin hydrogels containing epidermal growth factor (Gtn-EGF), when injected into the cavity after ICH, can support the brain tissue in rats, providing an innovative direction for antioxidant therapy and neural regeneration treatment after ICH [129].

Mesenchymal stem cell- (MSC-) based therapy had great potential application for ischemic stroke and was utilized in developing phase II trials in humans [130]. MSCs have obvious antioxidant properties that function through a variety of mechanisms, such as free radical scavenging, promotion of endogenous antioxidant defense, immune regulation by inhibiting ROS, changing the energy flow of mitochondria, and donating functional mitochondria to damaged cells [131]. Preclinical research has shown obvious benefits, but clinical studies had not observed obvious efficacy, which may be related to the death of MSCs caused by the environmental OS in transplantation [132, 133]. Nanomaterials can play a role in regulating the transplantation environment of MSCs and assist in the growth of neurons. After modifying human umbilical cord mesenchymal stem cells (HucMSCs) with hyaluronic acid-coated nanoceria, HucMSCs exerted significantly enhanced antioxidant capacities [134]. Nanoscaffolds and hydrogels can be used to encapsulate stem cells and provide conductive microenvironments for neural tissue regeneration [135, 136]. Moreover, nanostructured CeO_2_-loaded PLGA–ceramic scaffolds, MnO_2_ NP-dotted hydrogel (MnO_2_ nanoparticles in hyaluronic acid hydrogel), and core-shell hydrogel-loaded iron chelator agents (minocycline hydrochloride) have been found to allow more MSCs to survive, promoting cellular adhesion and support for MSC differentiation [135, 137, 138]. MSCs can be modified or encapsulated by nanomaterials to increase survival and promote neural regeneration.

## 4. Challenges and Prospect for Nanomaterials Application in the Treatment of Stroke

Nanomaterials have many advantages in the antioxidant application of stroke. At the same time, nanomaterials also have toxic effects. Nanomaterials can interact with compounds in cells, and they have cytotoxic effects that interfere with cell homeostasis. These cytotoxic effects are related to the size, shape, and surface properties of nanomaterials [139, 140]. The size of nanomaterials is an important factor affecting cytotoxicity. The smaller the nanomaterials, the greater the surface area to volume ratio, which allows them to react with a variety of chemical molecules within the cell, enhancing cytotoxicity [141]. The 5 nm AuNPs exhibit an oxidative stress-causing effect, as Au-NPs with a smaller diameter tend to accumulate in the nucleus and organelles, causing DNA damage [34, 142]. Polystyrene nanomaterials are changed from a sphere to a disk, with lower cell uptake and little impact on cell functions, such as cellular ROS generation [143]. Surface properties, such as chemical properties (hydrophobicity or hydrophilicity) and electrical properties (negatively charged or positive charged), are also aspects of nanotoxicity. Hydrophobic nanomaterials are more easily absorbed because of the presence of lipid membranes, so they are relatively more toxic. Similarly, the cell membrane is negatively charged. Therefore, positively charged nanomaterials are more easily absorbed than negatively charged nanomaterials [144–146]. The cytotoxicity is related to inflammatory reactions and ROS generation. For example, nanoceria cause proinflammatory cytokine production [147]. The cytotoxic effects limit the application of nanomaterials in the clinical setting, and it needs to find a compromised method to reduce cytotoxic effects by continuously changing the size, shape, and surface properties of nanomaterials.

Nanomaterials > 30 nm can be cleared by the MPS [46]. Moreover, they were cleared by the phagocytic cells in the liver and spleen, which may cause damage to the respective organs [148]. A study found that nanomaterials can be detected in the liver (40.04%), kidney (25.97%), brain (12.86%), heart, lungs, and spleen after oral administration of PLGA for 7 days [149]. Nanomaterials cause the adsorption of complement proteins and antibodies on their surfaces in blood called “corona,” act as signals to membrane receptors in immune cell, and induce phagocytosis [150, 151]. This decreased drug exposure and cerebral penetration causes nanomaterial accumulation in other organs besides the brain. Studies have found that nonionic, hydrophobic surfaces promote protein adsorption [152]. Thus, coating nanomaterials with hydrophilic polymers, such as PEG, can decrease MPS uptake, reduce immunogenicity, and prevent interactions with nontarget organs [153]. Moreover, as a result of higher ROS and H^+^ concentrations in the injured brain, nanomaterials that are pH/redox-responsive can achieve drug accumulation in ischemic tissue and decrease the dosage and off-target effects [154]. For example, the aryl oxalate can react with H_2_O_2_ to generate CO_2_ [155], copolyoxalate can be degraded into cyclohexanedimethanol and CO_2_ [156–158], and PLA is ROS-sensitive [30], and nanomaterial containing these substances can be pH/redox-responsive. The role and function of nanomaterials in other organs warrant evaluation, as methods to decrease off-target effects are related to the application of nanomaterials in clinical settings, which remain quite unclear and require further investigation.

Nanomaterials have promising clinical application; they are not used clinically in stroke. However, some researches indicated that nanomaterials have a big breakthrough in clinical application in stroke. Nanocurcumin has been used clinically in neurological diseases, such as amyotrophic lateral sclerosis (ALS) and multiple sclerosis (MS), showing antioxidant and immunity modulation [159–161]. Moreover, nanocurcumin demonstrates safety and tolerability in human subjects. However, there are possible side effects, such as abdominal pain [159]. It is believable that nanocurcumin can be studied clinically in stroke in the future. Although most nanomaterials appear to be harmless in preclinical trials, and their safety and tolerability in clinical application remain unknown. There are numerous demands for the use of nanomaterials in stroke antioxidant therapy. Firstly, the combined use of multiple antioxidants through various antioxidant pathways is a possible direction for antioxidant therapy in stroke, which poses a challenge to the drug-loading capacity of nanomaterials. Secondly, smart nanomaterial designs and physicochemical property studies are necessary to further increase the half-life and antioxidant effects of nanomaterials, decrease MPS resorption, and enhance suitability of nanomaterials for oral and intranasal administration. Lastly, many characteristics of nanomaterials used in antioxidant therapy, such as toxicity and off-targeted, require further elucidation in other animal models or organoids to promote clinical translation in stroke treatment and neural regeneration. The increasing incidence of stroke and the urgency of stroke antioxidant therapy will further promote the research of nanomaterials.

## 5. Conclusion

Nanomaterials are advanced biomaterials with controlled delivery of the antioxidants in stroke treatment and neural regeneration, suggesting a solution to overcome the lack of clinical translation. Some metals, metal oxides and carbon-based nanomaterials have antioxidant effects, which have been studied in numerous preclinical studies. The majority of studies have shown that nanomaterials deliver antioxidants to the brain at therapeutic doses with prolonged half-life, achieving greater therapeutic effects than free drugs. Nanomaterials improve the microenvironment after nerve injury and promote the survival of MSCs for poststroke repair. Nanomaterials possess promising biological applications and may address the current dilemma of antioxidant treatment in stroke.

## Figures and Tables

**Table 1:** Nanomaterials for the treatment of ischemia stroke-targeted oxidative stress.

Therapeutic molecules		Biomaterials and modifications	Main therapeutic effects	References
ROS scavenger nanoparticles	PtNPs	NPs	Reduce the infarct volume and the activation of MMP-9 and generation of •O^2-^	[56, 57]
	AuNPs	NPs	Reduce cerebral infarction, neuronal apoptosis, and oxidative stress	[34, 58]
	Nanoceria	NPs; PEG NPs; NPs encapsulated by ZIF-8	Downregulate iNOS; eliminate ONOO^−^; polarize microglia into M2 type	[59–61, 65]
	C60	NPs; hexasulfobutylated NPs	Activate the JNK pathway; inhibit cell apoptosis; reduce LDH release; increase NO	[41, 66]
	CNTs	PEG-SWCNT	Increase antioxidant enzymes	[44]
	PEG-HCC	PEG NPs	Exert functions as SOD; scavenge •OH; colocalize in the mitochondria	[67–69, 104]
Nanomaterials as carriers for ROS scavengers	TEMPO	RNPs; micelles	Reduced BBB damage; reduce the infarct size	[19, 70, 73]
	t-PA @ iRNP	Polyion composite NPs	Scavenges ROS and reduce the hemorrhage caused by t-PA	[74]
	Edaravone	Micelles; EA/P-CeO_2_	Improves outcome of ischemic stroke; shows synergistic scavenging activity of free radical	[20, 31]
	Vitamin E; vitamin C	Liposomes; PLGA NPs	Reduce the oxidative stress level	[45, 54]
	N-Acetylcysteine	Poly(amidoamine) dendrimer; PLGA NPs	Increases the antioxidant activity	[72, 75]
Nanomaterials as carriers for antioxidant enzymes	SOD	PLGA NPs; silica NPs coated TAT; nanoenzymes; PEI-PEG NPs; polyion complex NPs; silica NPs	Reduce the infarct area by 50% or more	[29, 55, 76–80]
Nanomaterials as carriers for antioxidants	Resveratrol	PVP-b-PCL NPs; MSNPs coated a ligand for LDLR; polymer NPs	Reduce the release of LDH and MDA content	[30, 83, 84]
	Quercetin	PLGA NPs	Reduce mitochondrial damage and ROS levels	[87]
	TNE	Carbitol chitosan NPs; PLGA-chitosan NPs	Improved the behavior; reduced lipid peroxidation	[85, 86]
	AKBA	Chitosan NPs	Increase the expression of Nrf2 and HO-1	[90]
	PNS	MPEG-PLGA NPs	Reduces the cerebral infarct volume by about 50%; reduces the concentration of H_2_O_2_ and MDA	[88]
	Lycopene	Liposomes	Reduces the levels of NOS and inhibit NOX2	[89]
	Gallic acid	Chitosan NPs	Reduce oxidative stress	[91]
	Retinoic acid	Polymeric NPs	Increase the proliferation and survival rate of endothelial cells	[92]
	EPO	Liposomes; PLGA NPs	Decrease the neurological deficits	[93–95]
Nanomaterial carriers for antioxidant genes	HO-1 gene	HSAP-NP; micelles; DA-PEI NPs; rPOA; PG2HR	Reduce oxidative stress	[32, 98–101]

Abbreviations: ROS: reactive oxygen species; NPs: nanoparticles; PtNPs: platinum nanoparticles; AuNPs: gold nanoparticles; nanoceria: cerium oxide nanoparticles; CNTs: carbon nanotubes; SWCNT: single-walled carbon nanotubes; HCC: hydrophilic carbon clusters; TEMPO: 2,2,6,6-tetramethylpiperidine-1-oxyl; t-PA @ iRNP: t-PA and 4-amino-TEMPO-containing self-assembled polyion composite nanoparticles; SOD: superoxide dismutase; TNE: thymoquinone nanoemulsion; AKBA: acetyl-11-keto-*β*-boswellic acid; PNS: Panax notoginseng; EPO: erythropoietin; HO-1: heme oxygenase-1; PEG: polyethylene glycol; ZIF-8: zeolitic imidazolate framework-8; PLGA: poly(lactic-co-glycolic acid); MPEG-PLGA: methyl ether PEG-PLGA; RNPs: nitroxide radical-containing nanoparticles; EA/P-CeO_2_: nanoceria loaded with edaravone and modified with PEG and angiopep-2; TAT: transactivator protein; nanoenzymes: SOD1 and methoxy-PEG-poly(L-lysine hydrochloride) chemically cross-linked; PEI: polyethyleneimine; PVP-b-PCL: poly(N-vinylpyrrolidone)-b-poly(*ε*-caprolactone) polymer; DA-PEI: deoxycholic acid-conjugated PEI; •O^2-^: superoxide anion; •OH: hydroxyl radicals; MMP-9: matrix metalloproteinase-9; iNOS: inducible nitric oxide synthase; ONOO^−^: peroxynitrite; LDH: lactate dehydrogenase; NO: nitric oxide; BBB: blood-brain barrier; MDA: malondialdehyde; Nrf2: nuclear factor-erythroid 2-related factor 2; NOX2: NADPH oxidase 2; JNK: c-Jun NH2 terminal protein kinase; rPOA: reducible poly(oligo-D-arginines); PG2HR: polyamidoamine generation 2 dendrimer conjugated histidine and arginine.

## Data Availability

The review data used to support the findings of this study are available from the corresponding author upon request.

## Abbreviations

ROS: Reactive oxygen species
BBB: Blood-brain barrier
PtNPs: Platinum nanoparticles
Nanoceria: Cerium oxide nanoparticles
MPS: Mononuclear phagocytic system
PEG: Polyethylene glycol
EPR: Enhanced permeability and retention
•O_2_^−^: Superoxide anion
•OH: Hydroxyl radicals
SOD: Superoxide dismutase
CAT: Catalase
CNTs: Carbon nanotubes
ELIP: Echogenic liposomes
PLGA: Poly(lactic-co-glycolic acid)
PLA: Polylactide
PAMAM: Poly(amidoamine)
PMMA: Poly(methyl methacrylate)
tMCAO: Transient middle cerebral artery occlusion
AuNPs: Gold nanoparticles
NO: Nitric oxide
ONOO^−^: Peroxynitrite
ZIF-8: Zeolitic imidazolate framework-8
OS: Oxidative stress
JNK: c-Jun NH2 terminal protein kinase
PARP: polyADP-ribose polymerase
LDH: Lactate dehydrogenase
SWCNT-PEG: Single-walled carbon nanotubes functionalized with polyethylene glycol
PEG-HCCs: PEGylated hydrophilic carbon clusters
TEMPO: 2,2,6,6-tetramethylpiperidine-1-oxyl
NAC: N-acetylcysteine
RNPs: Nitroxide radical-containing nanoparticles
t-PA @ iRNPs: t-PA and 4-amino-TEMPO-containing self-assembled polyion composite nanoparticles
HIV: Human immunodeficiency virus
TAT: Transactivator protein
PEI-PEG: Polyethyleneimine-polyethylene glycol
Nrf2: Nuclear factor-erythroid 2-related factor 2
PPAR*γ*: Peroxisome proliferator-activated receptor *γ*
FoxO: Forkhead box O
RES-NPs: Resveratrol-loaded nanoparticles
PVP-b-PCL: Poly (N-vinylpyrrolidone)-b-poly(*ε*-caprolactone) polymer
MSNP: Mesoporous silica nanoparticles
LDLR: Low-density lipoprotein receptor
PNS: Panax notoginseng
HLV: Hybrid liposomal vesicles
NOX2: NADPH oxidase 2
AUC: Area under the curve
AKBA-NP: Acetyl-11-keto-*β*-boswellic acid o-carboxymethyl chitosan nanoparticles
GA-NP: Gallic acid o-carboxymethyl chitosan nanoparticles
RA-NP: Retinoic acid nanoparticles
EPO: Erythropoietin
PTNPs: Platelet-mimetic nanoparticles
Syk: Spleen tyrosine kinase
DP2k: Deoxycholate-conjugated polyethylenimine-2k
RAGE: Receptor for advanced glycation end-products
HSAP: Hypoxia-specific anti-RAGE peptide
pHO1: Heme oxygenase-1 plasmid
DA-PEI: Deoxycholic acid-conjugated polyethyleneimine
rPOA: Reducible poly(oligo-D-arginines)
PG2: Polyamidoamine generation 2 dendrimers
ICH: Intracerebral hemorrhage
DEF: Deferoxamine
SAH: Subarachnoid hemorrhage
EBI: Early brain injury
CCM: Cerebral cavernous malformation
MEF cells: Mouse embryonic fibroblast cells
Pt5@Rapa NPs: Platinum@BSA-rapamycin nanoparticles
BSA: Bovine serum albumin
PEG-Se NPs: PEGylated Se nanoparticles
Gtn-EGF: Gelatin hydrogels containing epidermal growth factor
MSC: Mesenchymal stem cell
HucMSCs: Human umbilical cord mesenchymal stem cells
MnO_2_ NP-dotted hydrogel: MnO_2_ nanoparticles in hyaluronic acid hydrogel
ALS: Amyotrophic lateral sclerosis
MS: Multiple sclerosis

## References

[1] Benjamin E. J., Virani S. S., Callaway C. W. (2018). Heart disease and stroke Statistics-2018 update: a report from the American Heart Association. *Circulation*.

[2] GBD 2016 Lifetime Risk of Stroke Collaborators, Feigin V. L., Nguyen G. (2018). Global, regional, and country-specific lifetime risks of stroke, 1990 and 2016. *The New England Journal of Medicine*.

[3] Lin L., Wang X. (2016). Ischemia-reperfusion injury in the brain: mechanisms and potential therapeutic strategies. *Biochemistry & Pharmacology*.

[4] Duan X., Wen Z., Shen H., Shen M., Chen G. (2016). Intracerebral hemorrhage, oxidative stress, and antioxidant therapy. *Oxidative Medicine and Cellular Longevity*.

[5] Fujii M., Yan J., Rolland W. B., Soejima Y., Caner B., Zhang J. H. (2013). Early brain injury, an evolving frontier in subarachnoid hemorrhage research. *Translational Stroke Research*.

[6] Khoshnam S. E., Winlow W., Farzaneh M., Farbood Y., Moghaddam H. F. (2017). Pathogenic mechanisms following ischemic stroke. *Neurological Sciences*.

[7] Wu L., Xiong X., Wu X. (2020). Targeting oxidative stress and inflammation to prevent ischemia-reperfusion injury. *Frontiers in Molecular Neuroscience*.

[8] Chen H., Song Y. S., Chan P. H. (2009). Inhibition of NADPH oxidase is neuroprotective after ischemia-reperfusion. *Journal of Cerebral Blood Flow and Metabolism*.

[9] Kent T. A., Mandava P. (2016). Embracing biological and methodological variance in a new approach to pre-clinical stroke testing. *Translational Stroke Research*.

[10] Shuaib A., Lees K. R., Lyden P. (2007). NXY-059 for the treatment of acute ischemic stroke. *The New England Journal of Medicine*.

[11] Farokhzad O. C., Langer R. (2008). Impact of nanotechnology on drug delivery. *ACS Nano*.

[12] Kim K. S., Khang G., Lee D. (2011). Application of nanomedicine in cardiovascular diseases and stroke. *Current Pharmaceutical Design*.

[13] Chang E. H., Harford J. B., Eaton M. A. W. (2015). Nanomedicine: past, present and future - a global perspective. *Biochemical and Biophysical Research Communications*.

[14] Sandhir R., Yadav A., Sunkaria A., Singhal N. (2015). Nano-antioxidants: an emerging strategy for intervention against neurodegenerative conditions. *Neurochemistry International*.

[15] Narayanan K. B., Park H. H. (2013). Pleiotropic functions of antioxidant nanoparticles for longevity and medicine. *Advances in Colloid and Interface Science*.

[16] Moglianetti M., de Luca E., Pedone D. (2016). Platinum nanozymes recover cellular ROS homeostasis in an oxidative stress-mediated disease model. *Nanoscale*.

[17] Zou S., Zhu X., Zhang L. (2018). Biomineralization-inspired synthesis of cerium-doped carbonaceous nanoparticles for highly hydroxyl radical scavenging activity. *Nanoscale Research Letters*.

[18] Salatin S., Maleki Dizaj S., Yari Khosroushahi A. (2015). Effect of the surface modification, size, and shape on cellular uptake of nanoparticles. *Cell Biology International*.

[19] Hosoo H., Marushima A., Nagasaki Y. (2017). Neurovascular unit protection from cerebral ischemia-reperfusion injury by radical-containing nanoparticles in mice. *Stroke*.

[20] Jin Q., Cai Y., Li S. (2017). Edaravone-encapsulated agonistic micelles rescue ischemic brain tissue by tuning blood-brain barrier permeability. *Theranostics*.

[21] Mohammadi M. R., Nojoomi A., Mozafari M., Dubnika A., Inayathullah M., Rajadas J. (2017). Nanomaterials engineering for drug delivery: a hybridization approach. *Journal of Materials Chemistry B*.

[22] Saraiva C., Praça C., Ferreira R., Santos T., Ferreira L., Bernardino L. (2016). Nanoparticle-mediated brain drug delivery: overcoming blood-brain barrier to treat neurodegenerative diseases. *Journal of Controlled Release*.

[23] Suk J. S., Xu Q., Kim N., Hanes J., Ensign L. M. (2016). PEGylation as a strategy for improving nanoparticle-based drug and gene delivery. *Advanced Drug Delivery Reviews*.

[24] Pagels R. F., Prud'homme R. K. (2015). Polymeric nanoparticles and microparticles for the delivery of peptides, biologics, and soluble therapeutics. *Journal of Controlled Release*.

[25] Teleanu D. M., Chircov C., Grumezescu A., Volceanov A., Teleanu R. (2018). Blood-brain delivery methods using nanotechnology. *Pharmaceutics*.

[26] de Bock M., van Haver V., Vandenbroucke R. E., Decrock E., Wang N., Leybaert L. (2016). Into rather unexplored terrain-transcellular transport across the blood-brain barrier. *Glia*.

[27] Pardridge W. M. (2007). Blood-brain barrier delivery. *Drug Discovery Today*.

[28] Kim K. S., Song C. G., Kang P. M. (2019). Targeting oxidative stress using nanoparticles as a theranostic strategy for cardiovascular diseases. *Antioxidants & Redox Signaling*.

[29] Chen Y. P., Chen C. T., Hung Y. (2013). A new strategy for intracellular delivery of enzyme using mesoporous silica nanoparticles: superoxide dismutase. *Journal of the American Chemical Society*.

[30] Shen Y., Cao B., Snyder N. R., Woeppel K. M., Eles J. R., Cui X. T. (2018). ROS responsive resveratrol delivery from LDLR peptide conjugated PLA-coated mesoporous silica nanoparticles across the blood-brain barrier. *Journal of Nanobiotechnology*.

[31] Bao Q., Hu P., Xu Y. (2018). Simultaneous blood-brain barrier crossing and protection for stroke treatment based on edaravone-loaded ceria nanoparticles. *ACS Nano*.

[32] Oh J., Lee J., Piao C., Jeong J. H., Lee M. (2019). A self-assembled DNA-nanoparticle with a targeting peptide for hypoxia-inducible gene therapy of ischemic stroke. *Biomaterials Science*.

[33] Zhang T. T., Li W., Meng G., Wang P., Liao W. (2016). Strategies for transporting nanoparticles across the blood-brain barrier. *Biomaterials Science*.

[34] Zheng Y., Wu Y., Liu Y. (2019). Intrinsic effects of gold nanoparticles on oxygen-glucose deprivation/reperfusion injury in rat cortical neurons. *Neurochemical Research*.

[35] Celardo I., Pedersen J. Z., Traversa E., Ghibelli L. (2011). Pharmacological potential of cerium oxide nanoparticles. *Nanoscale*.

[36] Pedone D., Moglianetti M., de Luca E., Bardi G., Pompa P. P. (2017). Platinum nanoparticles in nanobiomedicine. *Chemical Society Reviews*.

[37] Kim C. K., Kim T., Choi I. Y. (2012). Ceria nanoparticles that can protect against ischemic stroke. *Angewandte Chemie (International Ed. in English)*.

[38] Jeong H. G., Cha B. G., Kang D. W. (2018). Ceria nanoparticles synthesized with aminocaproic acid for the treatment of subarachnoid hemorrhage. *Stroke*.

[39] Dellinger A., Zhou Z., Connor J. (2013). Application of fullerenes in nanomedicine: an update. *Nanomedicine (London, England)*.

[40] Foley S., Crowley C., Smaihi M. (2002). Cellular localisation of a water-soluble fullerene derivative. *Biochemical and Biophysical Research Communications*.

[41] Lao F., Chen L., Li W. (2009). Fullerene nanoparticles selectively enter oxidation-damaged cerebral microvessel endothelial cells and inhibit JNK-related apoptosis. *ACS Nano*.

[42] Amani H., Habibey R., Hajmiresmail S. J., Latifi S., Pazoki-Toroudi H., Akhavan O. (2017). Antioxidant nanomaterials in advanced diagnoses and treatments of ischemia reperfusion injuries. *Journal of Materials Chemistry B*.

[43] Vardharajula S., Ali S. Z., Tiwari P. M. (2012). Functionalized carbon nanotubes: biomedical applications. *International Journal of Nanomedicine*.

[44] Dal Bosco L., Weber G. E., Parfitt G. M. (2015). Biopersistence of PEGylated carbon nanotubes promotes a delayed antioxidant response after infusion into the rat hippocampus. *PLoS One*.

[45] Sinha J., Das N., Basu M. K. (2001). Liposomal antioxidants in combating ischemia-reperfusion injury in rat brain. *Biomedicine & Pharmacotherapy*.

[46] Malam Y., Loizidou M., Seifalian A. M. (2009). Liposomes and nanoparticles: nanosized vehicles for drug delivery in cancer. *Trends in Pharmacological Sciences*.

[47] Panahi Y., Farshbaf M., Mohammadhosseini M. (2017). Recent advances on liposomal nanoparticles: synthesis, characterization and biomedical applications. *Artificial Cells, Nanomedicine, and Biotechnology*.

[48] Lakkadwala S., Singh J. (2019). Co-delivery of doxorubicin and erlotinib through liposomal nanoparticles for glioblastoma tumor regression using an in vitro brain tumor model. *Colloids and Surfaces. B, Biointerfaces*.

[49] Kim H., Britton G. L., Peng T., Holland C. K., McPherson D., Huang S. L. (2014). Nitric oxide-loaded echogenic liposomes for treatment of vasospasm following subarachnoid hemorrhage. *International Journal of Nanomedicine*.

[50] Crucho C. I. C., Barros M. T. (2017). Polymeric nanoparticles: a study on the preparation variables and characterization methods. *Materials Science & Engineering. C, Materials for Biological Applications*.

[51] El-Say K. M., El-Sawy H. S. (2017). Polymeric nanoparticles: promising platform for drug delivery. *International Journal of Pharmaceutics*.

[52] Cabral H., Kataoka K. (2014). Progress of drug-loaded polymeric micelles into clinical studies. *Journal of Controlled Release*.

[53] Yokoyama M. (2014). Polymeric micelles as drug carriers: their lights and shadows. *Journal of Drug Targeting*.

[54] Astete C. E., Dolliver D., Whaley M., Khachatryan L., Sabliov C. M. (2011). Antioxidant poly(lactic-co-glycolic) acid nanoparticles made with *α*-tocopherol-ascorbic acid surfactant. *ACS Nano*.

[55] Reddy M. K., Labhasetwar V. (2009). Nanoparticle-mediated delivery of superoxide dismutase to the brain: an effective strategy to reduce ischemia-reperfusion injury. *The FASEB Journal*.

[56] Takamiya M., Miyamoto Y., Yamashita T. (2011). Neurological and pathological improvements of cerebral infarction in mice with platinum nanoparticles. *Journal of Neuroscience Research*.

[57] Takamiya M., Miyamoto Y., Yamashita T., Deguchi K., Ohta Y., Abe K. (2012). Strong neuroprotection with a novel platinum nanoparticle against ischemic stroke- and tissue plasminogen activator-related brain damages in mice. *Neuroscience*.

[58] Liu Z., Shen Y., Wu Y. (2013). An intrinsic therapy of gold nanoparticles in focal cerebral ischemia-reperfusion injury in rats. *Journal of Biomedical Nanotechnology*.

[59] Estevez A. Y., Pritchard S., Harper K. (2011). Neuroprotective mechanisms of cerium oxide nanoparticles in a mouse hippocampal brain slice model of ischemia. *Free Radical Biology & Medicine*.

[60] Hirst S. M., Karakoti A. S., Tyler R. D., Sriranganathan N., Seal S., Reilly C. M. (2009). Anti-inflammatory properties of cerium oxide nanoparticles. *Small*.

[61] Dowding J. M., Seal S., Self W. T. (2013). Cerium oxide nanoparticles accelerate the decay of peroxynitrite (ONOO(-)). *Drug Delivery and Translational Research*.

[62] Zeng F., Wu Y., Li X. (2018). Custom-made ceria nanoparticles show a neuroprotective effect by modulating phenotypic polarization of the microglia. *Angewandte Chemie (International Ed. in English)*.

[63] Clark A., Zhu A., Sun K., Petty H. R. (2011). Cerium oxide and platinum nanoparticles protect cells from oxidant-mediated apoptosis. *Journal of Nanoparticle Research*.

[64] Weidinger A., Kozlov A. V. (2015). Biological activities of reactive oxygen and nitrogen species: oxidative stress versus signal transduction. *Biomolecules*.

[65] He L., Huang G., Liu H., Sang C., Liu X., Chen T. (2020). Highly bioactive zeolitic imidazolate framework-8-capped nanotherapeutics for efficient reversal of reperfusion-induced injury in ischemic stroke. *Science Advances*.

[66] Huang S. S., Tsai S. K., Chih C. L. (2001). Neuroprotective effect of hexasulfobutylated C_60_ on rats subjected to focal cerebral ischemia. *Free Radical Biology & Medicine*.

[67] Fabian R. H., Derry P. J., Rea H. C. (2018). Efficacy of novel carbon nanoparticle antioxidant therapy in a severe model of reversible middle cerebral artery stroke in acutely hyperglycemic rats. *Frontiers in Neurology*.

[68] Samuel E. L., Marcano D. C., Berka V. (2015). Highly efficient conversion of superoxide to oxygen using hydrophilic carbon clusters. *Proceedings of the National Academy of Sciences of the United States of America*.

[69] Derry P. J., Nilewski L. G., Sikkema W. K. A. (2019). Catalytic oxidation and reduction reactions of hydrophilic carbon clusters with NADH and cytochrome C: features of an electron transport nanozyme. *Nanoscale*.

[70] Marushima A., Suzuki K., Nagasaki Y. (2011). Newly synthesized radical-containing nanoparticles enhance neuroprotection after cerebral ischemia-reperfusion injury. *Neurosurgery*.

[71] Zhang D., Xiao Y., Lv P. (2017). Edaravone attenuates oxidative stress induced by chronic cerebral hypoperfusion injury: role of ERK/Nrf2/HO-1 signaling pathway. *Neurological Research*.

[72] Kurtoglu Y. E., Navath R. S., Wang B., Kannan S., Romero R., Kannan R. M. (2009). Poly(amidoamine) dendrimer-drug conjugates with disulfide linkages for intracellular drug delivery. *Biomaterials*.

[73] Yoshitomi T., Nagasaki Y. (2011). Nitroxyl radical-containing nanoparticles for novel nanomedicine against oxidative stress injury. *Nanomedicine (London, England)*.

[74] Mei T., Kim A., Vong L. B. (2019). Encapsulation of tissue plasminogen activator in pH-sensitive self-assembled antioxidant nanoparticles for ischemic stroke treatment - Synergistic effect of thrombolysis and antioxidant -. *Biomaterials*.

[75] Karimi Zarchi A. A., Abbasi S., Faramarzi M. A., Gilani K., Ghazi-Khansari M., Amani A. (2015). Development and optimization of N-Acetylcysteine-loaded poly (lactic-co- glycolic acid) nanoparticles by electrospray. *International Journal of Biological Macromolecules*.

[76] Petro M., Jaffer H., Yang J., Kabu S., Morris V. B., Labhasetwar V. (2016). Tissue plasminogen activator followed by antioxidant-loaded nanoparticle delivery promotes activation/mobilization of progenitor cells in infarcted rat brain. *Biomaterials*.

[77] Manickam D. S., Brynskikh A. M., Kopanic J. L. (2012). Well-defined cross-linked antioxidant nanozymes for treatment of ischemic brain injury. *Journal of Controlled Release*.

[78] Rosenbaugh E. G., Roat J. W., Gao L. (2010). The attenuation of central angiotensin II-dependent pressor response and intra-neuronal signaling by intracarotid injection of nanoformulated copper/zinc superoxide dismutase. *Biomaterials*.

[79] Jiang Y., Brynskikh A. M., S-Manickam D., Kabanov A. V. (2015). SOD1 nanozyme salvages ischemic brain by locally protecting cerebral vasculature. *Journal of Controlled Release*.

[80] Ambati J., Lopez A. M., Cochran D. (2012). Engineered silica nanocarriers as a high-payload delivery vehicle for antioxidant enzymes. *Acta Biomaterialia*.

[81] Cheng Y. C., Sheen J. M., Hu W. L., Hung Y. C. (2017). Polyphenols and oxidative stress in atherosclerosis-related ischemic heart disease and stroke. *Oxidative Medicine and Cellular Longevity*.

[82] Pallauf K., Duckstein N., Hasler M., Klotz L. O., Rimbach G. (2017). Flavonoids as putative inducers of the transcription factors Nrf2, FoxO, and PPAR*γ*. *Oxidative Medicine and Cellular Longevity*.

[83] Lu X., Xu H., Sun B., Zhu Z., Zheng D., Li X. (2013). Enhanced neuroprotective effects of resveratrol delivered by nanoparticles on hydrogen peroxide-induced oxidative stress in rat cortical cell culture. *Molecular Pharmaceutics*.

[84] Lu X., Dong J., Zheng D., Li X., Ding D., Xu H. (2020). Reperfusion combined with intraarterial administration of resveratrol-loaded nanoparticles improved cerebral ischemia-reperfusion injury in rats. *Nanomedicine*.

[85] Ahmad N., Ahmad R., Alam M. A., Samim M., Iqbal Z., Ahmad F. J. (2016). Quantification and evaluation of thymoquinone loaded mucoadhesive nanoemulsion for treatment of cerebral ischemia. *International Journal of Biological Macromolecules*.

[86] Xiao X. Y., Zhu Y. X., Bu J. Y., Li G. W., Zhou J. H., Zhou S. P. (2016). Evaluation of neuroprotective effect of thymoquinone nanoformulation in the rodent cerebral ischemia-reperfusion model. *BioMed Research International*.

[87] Ghosh A., Sarkar S., Mandal A. K., Das N. (2013). Neuroprotective role of nanoencapsulated quercetin in combating ischemia-reperfusion induced neuronal damage in young and aged rats. *PLoS One*.

[88] Zhang J., Han X., Li X. (2012). Core-shell hybrid liposomal vesicles loaded with panax notoginsenoside: preparation, characterization and protective effects on global cerebral ischemia/reperfusion injury and acute myocardial ischemia in rats. *International Journal of Nanomedicine*.

[89] Zhao Y., Xin Z., Li N. (2018). Nano-liposomes of lycopene reduces ischemic brain damage in rodents by regulating iron metabolism. *Free Radical Biology & Medicine*.

[90] Ding Y., Qiao Y., Wang M. (2016). Enhanced neuroprotection of acetyl-11-keto-*β*-boswellic acid (AKBA)-loaded O-carboxymethyl chitosan nanoparticles through antioxidant and anti-inflammatory pathways. *Molecular Neurobiology*.

[91] Zhao Y., Li D., Zhu Z., Sun Y. (2020). Improved neuroprotective effects of gallic acid-loaded chitosan nanoparticles against ischemic stroke. *Rejuvenation Research*.

[92] Machado-Pereira M., Santos T., Ferreira L., Bernardino L., Ferreira R. (2018). Intravenous administration of retinoic acid-loaded polymeric nanoparticles prevents ischemic injury in the immature brain. *Neuroscience Letters*.

[93] Ishii T., Asai T., Fukuta T. (2012). A single injection of liposomal asialo-erythropoietin improves motor function deficit caused by cerebral ischemia/reperfusion. *International Journal of Pharmaceutics*.

[94] Ishii T., Asai T., Oyama D. (2012). Amelioration of cerebral ischemia-reperfusion injury based on liposomal drug delivery system with asialo-erythropoietin. *Journal of Controlled Release*.

[95] Chen H., Spagnoli F., Burris M. (2012). Nanoerythropoietin is 10-times more effective than regular erythropoietin in neuroprotection in a neonatal rat model of hypoxia and ischemia. *Stroke*.

[96] Cai W., Liu S., Hu M. (2020). Functional dynamics of neutrophils after ischemic stroke. *Translational Stroke Research*.

[97] Tang C., Wang C., Zhang Y. (2019). Recognition, intervention, and monitoring of neutrophils in acute ischemic stroke. *Nano Letters*.

[98] Lee J., Hyun H., Kim J. (2012). Dexamethasone-loaded peptide micelles for delivery of the heme oxygenase-1 gene to ischemic brain. *Journal of Controlled Release*.

[99] Oh J., Lee M. S., Jeong J. H., Lee M. (2017). Deoxycholic acid-conjugated polyethylenimine for delivery of heme oxygenase-1 gene in rat ischemic stroke model. *Journal of Pharmaceutical Sciences*.

[100] Hyun H., Won Y. W., Kim K. M., Lee J., Lee M., Kim Y. H. (2010). Therapeutic effects of a reducible poly (oligo-D-arginine) carrier with the heme oxygenase-1 gene in the treatment of hypoxic-ischemic brain injury. *Biomaterials*.

[101] Lee Y., Lee J., Kim M., Kim G., Choi J. S., Lee M. (2021). Brain gene delivery using histidine and arginine-modified dendrimers for ischemic stroke therapy. *Journal of Controlled Release*.

[102] Selim M., Foster L. D., Moy C. S. (2019). Deferoxamine mesylate in patients with intracerebral haemorrhage (i-DEF): a multicentre, randomised, placebo-controlled, double-blind phase 2 trial. *Lancet Neurology*.

[103] Dharmalingam P., Talakatta G., Mitra J. (2020). Pervasive genomic damage in experimental intracerebral hemorrhage: therapeutic potential of a mechanistic-based carbon nanoparticle. *ACS Nano*.

[104] Galho A. R., Cordeiro M. F., Ribeiro S. A. (2016). Protective role of free and quercetin-loaded nanoemulsion against damage induced by intracerebral haemorrhage in rats. *Nanotechnology*.

[105] Connolly E. S., Rabinstein A. A., Carhuapoma J. R. (2012). Guidelines for the management of aneurysmal subarachnoid hemorrhage: a guideline for healthcare professionals from the American Heart Association/american Stroke Association. *Stroke*.

[106] Fumoto T., Naraoka M., Katagai T., Li Y., Shimamura N., Ohkuma H. (2019). The role of oxidative stress in microvascular disturbances after experimental subarachnoid hemorrhage. *Translational Stroke Research*.

[107] Chang C. Z., Wu S. C., Lin C. L., Kwan A. L. (2015). Curcumin, encapsulated in nano-sized PLGA, down-regulates nuclear factor *κ*B (p65) and subarachnoid hemorrhage induced early brain injury in a rat model. *Brain Research*.

[108] Kim M. W., An S., Kim K. (2018). Packing of metalized polymer nanofibers for aneurysm embolization. *Nanoscale*.

[109] Batra S., Lin D., Recinos P. F., Zhang J., Rigamonti D. (2009). Cavernous malformations: natural history, diagnosis and treatment. *Nature Reviews. Neurology*.

[110] McKerracher L., Shenkar R., Abbinanti M. (2020). A brain-targeted orally available ROCK2 inhibitor benefits mild and aggressive cavernous angioma disease. *Translational Stroke Research*.

[111] Retta S. F., Glading A. J. (2016). Oxidative stress and inflammation in cerebral cavernous malformation disease pathogenesis: two sides of the same coin. *International Journal of Biochemistry & Cell Biology*.

[112] Marchi S., Corricelli M., Trapani E. (2015). Defective autophagy is a key feature of cerebral cavernous malformations. *EMBO Molecular Medicine*.

[113] De Luca E., Pedone D., Moglianetti M. (2018). Multifunctional platinum@BSA-rapamycin nanocarriers for the combinatorial therapy of cerebral cavernous malformation. *ACS Omega*.

[114] Gerhke S. A., Shibli J. A., Salles M. B. (2015). Potential of the use of an antioxidant compound to promote peripheral nerve regeneration after injury. *Neural Regeneration Research*.

[115] Koh H. S., Yong T., Chan C. K., Ramakrishna S. (2008). Enhancement of neurite outgrowth using nano-structured scaffolds coupled with laminin. *Biomaterials*.

[116] Lim T. C., Spector M. (2017). Biomaterials for enhancing CNS repair. *Translational Stroke Research*.

[117] Baranes K., Shevach M., Shefi O., Dvir T. (2016). Gold nanoparticle-decorated scaffolds promote neuronal differentiation and maturation. *Nano Letters*.

[118] Ciofani G., Genchi G. G., Liakos I. (2013). Effects of cerium oxide nanoparticles on PC12 neuronal-like cells: proliferation, differentiation, and dopamine secretion. *Pharmaceutical Research*.

[119] Marino A., Tonda-Turo C., de Pasquale D. (2017). Gelatin/nanoceria nanocomposite fibers as antioxidant scaffolds for neuronal regeneration. *Biochimica et Biophysica Acta - General Subjects*.

[120] Das M., Patil S., Bhargava N. (2007). Auto-catalytic ceria nanoparticles offer neuroprotection to adult rat spinal cord neurons. *Biomaterials*.

[121] Gliga A. R., Edoff K., Caputo F. (2017). Cerium oxide nanoparticles inhibit differentiation of neural stem cells. *Scientific Reports*.

[122] Pulido-Reyes G., Rodea-Palomares I., Das S. (2015). Untangling the biological effects of cerium oxide nanoparticles: the role of surface valence states. *Scientific Reports*.

[123] Naganuma T., Traversa E. (2014). The effect of cerium valence states at cerium oxide nanoparticle surfaces on cell proliferation. *Biomaterials*.

[124] Lovat V., Pantarotto D., Lagostena L. (2005). Carbon nanotube substrates boost neuronal electrical signaling. *Nano Letters*.

[125] Lewitus D. Y., Landers J., Branch J. R. (2011). Biohybrid carbon nanotube/agarose fibers for neural tissue engineering. *Advanced Functional Materials*.

[126] Chao T. I., Xiang S., Chen C. S. (2009). Carbon nanotubes promote neuron differentiation from human embryonic stem cells. *Biochemical and Biophysical Research Communications*.

[127] Amani H., Habibey R., Shokri F. (2019). Selenium nanoparticles for targeted stroke therapy through modulation of inflammatory and metabolic signaling. *Scientific Reports*.

[128] Wang J., Tian L., Luo B. (2018). Engineering PCL/lignin nanofibers as an antioxidant scaffold for the growth of neuron and Schwann cell. *Colloids and Surfaces. B, Biointerfaces*.

[129] Lim T. C., Mandeville E., Weng D. (2020). Hydrogel-based therapy for brain repair after intracerebral hemorrhage. *Translational Stroke Research*.

[130] Deng L., Peng Q., Wang H. (2019). Intrathecal injection of allogenic bone marrow-derived mesenchymal stromal cells in treatment of patients with severe ischemic stroke: study protocol for a randomized controlled observer-blinded trial. *Translational Stroke Research*.

[131] Stavely R., Nurgali K. (2020). The emerging antioxidant paradigm of mesenchymal stem cell therapy. *Stem Cells Translational Medicine*.

[132] Lalu M. M., Montroy J., Dowlatshahi D. (2020). From the lab to patients: a systematic review and meta-analysis of mesenchymal stem cell therapy for stroke. *Translational Stroke Research*.

[133] for the ISIS-HERMES Study Group, Jaillard A., Hommel M. (2020). Autologous mesenchymal stem cells improve motor recovery in subacute ischemic stroke: a randomized clinical trial. *Translational Stroke Research*.

[134] Zuo L., Feng Q., Han Y. (2019). Therapeutic effect on experimental acute cerebral infarction is enhanced after nanoceria labeling of human umbilical cord mesenchymal stem cells. *Therapeutic Advances in Neurological Disorders*.

[135] Mandoli C., Pagliari F., Pagliari S. (2010). Stem cell aligned growth induced by CeO2 nanoparticles in PLGA scaffolds with improved bioactivity for regenerative medicine. *Advanced Functional Materials*.

[136] Gopalakrishnan A., Shankarappa S. A., Rajanikant G. K. (2019). Hydrogel scaffolds: towards restitution of ischemic stroke-injured brain. *Translational Stroke Research*.

[137] Li L., Xiao B., Mu J. (2019). A MnO(2) nanoparticle-dotted hydrogel promotes spinal cord repair via rregulating reactive oxygen species microenvironment and synergizing with mesenchymal stem cells. *ACS Nano*.

[138] Gong Y., Wang Y., Qu Q. (2020). Nanoparticle encapsulated core-shell hydrogel for on-site BMSCs delivery protects from iron overload and enhances functional recovery. *Journal of Controlled Release*.

[139] De Matteis V., Rinaldi R. (2018). Toxicity assessment in the nanoparticle era. *Advances in Experimental Medicine and Biology*.

[140] Mu Q., Jiang G., Chen L. (2014). Chemical basis of interactions between engineered nanoparticles and biological systems. *Chemical Reviews*.

[141] Xia Q., Li H., Liu Y., Zhang S., Feng Q., Xiao K. (2017). The effect of particle size on the genotoxicity of gold nanoparticles. *Journal of Biomedical Materials Research. Part A*.

[142] Lopez-Chaves C., Soto-Alvaredo J., Montes-Bayon M., Bettmer J., Llopis J., Sanchez-Gonzalez C. (2018). Gold nanoparticles: Distribution, bioaccumulation and toxicity. _In vitro_ and _in vivo_ studies. *Nanomedicine*.

[143] Zhang Y., Tekobo S., Tu Y. (2012). Permission to enter cell by shape: nanodisk vs nanosphere. *ACS Applied Materials & Interfaces*.

[144] Brkić Ahmed L., Milić M., Pongrac I. M. (2017). Impact of surface functionalization on the uptake mechanism and toxicity effects of silver nanoparticles in HepG2 cells. *Food and Chemical Toxicology*.

[145] el Badawy A. M., Silva R. G., Morris B., Scheckel K. G., Suidan M. T., Tolaymat T. M. (2011). Surface charge-dependent toxicity of silver nanoparticles. *Environmental Science & Technology*.

[146] Cho E. C., Xie J., Wurm P. A., Xia Y. (2009). Understanding the role of surface charges in cellular adsorption versus internalization by selectively removing gold nanoparticles on the cell surface with a I2/KI etchant. *Nano Letters*.

[147] Wingard C. J., Walters D. M., Cathey B. L. (2010). Mast cells contribute to altered vascular reactivity and ischemia-reperfusion injury following cerium oxide nanoparticle instillation. *Nanotoxicology*.

[148] Lin Z., Monteiro-Riviere N. A., Riviere J. E. (2015). Pharmacokinetics of metallic nanoparticles. *Wiley Interdisciplinary Reviews. Nanomedicine and Nanobiotechnology*.

[149] Semete B., Booysen L., Lemmer Y. (2010). In vivo evaluation of the biodistribution and safety of PLGA nanoparticles as drug delivery systems. *Nanomedicine*.

[150] Monopoli M. P., Walczyk D., Campbell A. (2011). Physical-chemical aspects of protein corona: relevance to in vitro and in vivo biological impacts of nanoparticles. *Journal of the American Chemical Society*.

[151] Lynch I., Cedervall T., Lundqvist M., Cabaleiro-Lago C., Linse S., Dawson K. A. (2007). The nanoparticle-protein complex as a biological entity; a complex fluids and surface science challenge for the 21st century. *Advances in Colloid and Interface Science*.

[152] Moghimi S. M., Hunter A. C., Murray J. C. (2001). Long-circulating and target-specific nanoparticles: theory to practice. *Pharmacological Reviews*.

[153] Chapman A. P. (2002). PEGylated antibodies and antibody fragments for improved therapy: a review. *Advanced Drug Delivery Reviews*.

[154] Kwon J., Kim J., Park S., Khang G., Kang P. M., Lee D. (2013). Inflammation-responsive antioxidant nanoparticles based on a polymeric prodrug of vanillin. *Biomacromolecules*.

[155] Tapeinos C., Pandit A. (2016). Physical, chemical, and biological structures based on ROS-sensitive moieties that are able to respond to oxidative microenvironments. *Advanced Materials*.

[156] Lee D., Hong D., Lim H. (2013). H_2_O_2_-responsive molecularly engineered polymer nanoparticles as ischemia/reperfusion-targeted nanotherapeutic agents. *Scientific Reports*.

[157] Lee D., Bae S., Ke Q. (2013). Hydrogen peroxide-responsive copolyoxalate nanoparticles for detection and therapy of ischemia-reperfusion injury. *Journal of Controlled Release*.

[158] Park H., Kim S., Kim S. (2010). Antioxidant and anti-inflammatory activities of hydroxybenzyl alcohol releasing biodegradable polyoxalate nanoparticles. *Biomacromolecules*.

[159] Ahmadi M., Agah E., Nafissi S. (2018). Safety and efficacy of nanocurcumin as add-on therapy to riluzole in patients with amyotrophic lateral sclerosis: a pilot randomized clinical trial. *Neurotherapeutics*.

[160] Dolati S., Babaloo Z., Ayromlou H. (2019). Nanocurcumin improves regulatory T-cell frequency and function in patients with multiple sclerosis. *Journal of Neuroimmunology*.

[161] Karthikeyan A., Senthil N., Min T. (2020). Nanocurcumin: a promising candidate for therapeutic applications. *Frontiers in Pharmacology*.

